# Anatomical Factors Affecting the Complexity of Maxillary Sinus Augmentation in Saudi Patients: A Cone Beam Computed Tomography (CBCT) Study

**DOI:** 10.7759/cureus.68462

**Published:** 2024-09-02

**Authors:** Salwa Aldahlawi, Dalia Nourah, Ehdaa Alturkistani, Wejdan AlBander, Raneem Y Azab

**Affiliations:** 1 Department of Basic and Clinical Oral Science, Faculty of Dental Medicine, Umm Al-Qura University, Makkah, SAU; 2 Department of Periodontics, Faculty of Dental Medicine, Umm Al-Qura University, Makkah, SAU; 3 Department of Dentistry, Umm Al-Qura University, Makkah, SAU

**Keywords:** aaa, cone-beam conventional tomography (cbct), residual alveolar ridge height, sinus septa, maxillary sinus anatomy

## Abstract

Aim: This study aims to evaluate the anatomical variations of the maxillary sinus and determine the difficulty score of maxillary sinus augmentation (MSA) in Saudi patients seeking dental implant rehabilitation of the posterior maxilla using cone beam computed tomography (CBCT).

Methodology: CBCT records of dental patients seeking treatment at a University Dental Teaching Hospital between 2019 and 2023 were retrospectively analyzed. Measurements were obtained from CBCT images, including membrane thickness, sinus width, buccal bone thickness, presence of maxillary sinus septa, residual alveolar ridge height, angle of the buccolingual sinus wall, and the presence of the alveolar antral artery (AAA). The difficulty score for MSA was determined based on these anatomical factors.

Results: A total of 107 maxillary sinuses in 86 subjects were evaluated. The average membrane thickness was 2.23 mm, with males showing significantly higher thickness than females. Sinus septa were found in 54 (50.5%) sinuses, with 18 (17%) of sinuses having interfering septa. Twenty-three (21.5%) sinuses had a residual alveolar ridge height of less than 4 mm. The average angle of the buccolingual sinus wall was 79.39°, indicating a high prevalence of wide-shaped sinuses. The average sinus width was 14.09 mm, with 55 sinuses (51.4%) less than 15 mm. The average buccal bone thickness was 1.07 mm, in 29 (27%) sinuses, the thickness was more than 2 mm. AAA was visualized in 60 (56%) of sinuses, with 45 (42%) of sinuses having AAA interfering with the MSA window.

Conclusions: In this study, most sinuses were classified as simple or moderate difficulty, with higher membrane thickness, presence of septa, and AAA being the significant risk factors for complications.

These findings provide valuable insights for implant surgeons in Saudi patients seeking dental implant rehabilitation of the posterior maxilla, enabling them to anticipate and minimize potential complications during MSA procedures.

## Introduction

Maxillary sinus pneumatization and alveolar bone resorption are considered consequences of long-term edentulism [1]. These changes complicate dental implant placement in the posterior maxilla by decreasing the bone volume available for successful integration of the dental implant [2,3]. Maxillary sinus augmentation (MSA) involves Schneiderian membrane elevation to increase the residual bone height, thereby increasing the bone volume needed for the placement of dental implants [3]. Lateral window sinus augmentation is composed of the following steps: elevation of a mucoperiosteal flap, creating a boney window to access the sinus cavity, elevating the Schneiderian membrane above the maxillary floor to create a closed confined space bounded by the alveolar ridge, lateral wall of the sinus and the Schneider membrane. A bone graft may or may not be used to add volume to the area and the surgical site is closed and allowed for undisturbed healing [4].

MSA is considered a predictable procedure with an implant survival rate of 96.9% [5]. However, this surgical intervention is associated with increased morbidity and patient discomfort [2]. Perforation of the Schneiderian membrane is the most frequently reported complication of MSA, with an average rate of 30 % [6,7]. An intact Schneiderian membrane is detrimental to maintaining the confined space and maximizing the osteogenic potential of the site. Membrane perforation has been associated with graft infection, loss, and implant failure [8]. Perforation occurs when the tension applied exceeds the stretching potential of the membrane, which is closely related to the membrane’s health and thickness. Various anatomical characteristics such as maxillary sinus width and contour [9] and the presence and direction of maxillary septa have played a significant role in increasing the incidence of membrane perforation [10, 11].

The second most common complication of MSA is alveolar antral artery (AAA) injury during lateral window osteotomy [12, 13]. Anatomically, AAA arises from anastomosis between the posterior superior alveolar artery and infra-orbital artery and is always found at the lateral antral wall [14]. In 30% of the patients, the AAA is present at the site of lateral window osteotomy [15]. Artery damage during osteotomy leads to perfuse bleeding, and the membrane perforation rate increases due to lack of visibility [13].

There are several keys to minimizing complications during any surgical procedure. First, the clinician must have adequate knowledge of the regional and surgical anatomy and anatomical variations affected by genetic and racial variability [4]. Secondly, appropriate diagnostic and investigational methods must be utilized. In implant dentistry, the use of cone beam CT (CBCT) is mandatory during the treatment planning steps. CBCT allows the visualization of anatomical factors that could complicate the MSA [16]. Recognizing the risk factors preoperatively helps to identify solutions to potential problems and allows for precautionary measures that lead to the proper execution of the plan.

This study aims to determine the anatomical variants of the maxillary sinus and the difficulty score of MSA in Saudi patients seeking dental implant rehabilitation of the posterior maxilla by using CBCT.

## Materials and methods

Study design 

A retrospective study was conducted using CBCT records of dental patients seeking treatment at a University Dental Teaching Hospital between 2019 and 2023. Ethical approval was obtained from the Biomedical Research Ethics Committee at Umm Al-Qura University (Approval no. HAPO-02-K-2021-10-809). CBCT scans of adults above the age of 18 years with at least one first or second molar missing were included. CBCT Images that were unclear due to scattering or other reasons, previously grafted sinuses or ones with implants had been placed, and those with sinus pathology (such as polypoid lesions, sinus opacification, tumors, and perforation) that may compromise the measurement were excluded.

CBCT images

The CBCT were obtained using a Cone Beam system (i-CAT Vision Q System) set at 120 kVp and 37.07 mAs, with acquisition time 26.9 sec, and assessed using ICAT Vision viewer (version 1.9.3.13) Scans with 0.25 mm slices in the axial, coronal, and sagittal planes were used to obtain the measurement.

CBCT analysis and assessment

All measurements were performed in millimeters (mm) and obtained from the center of the edentulous area (representing the future implant site).

Membrane Thickness

**Figure 1:**
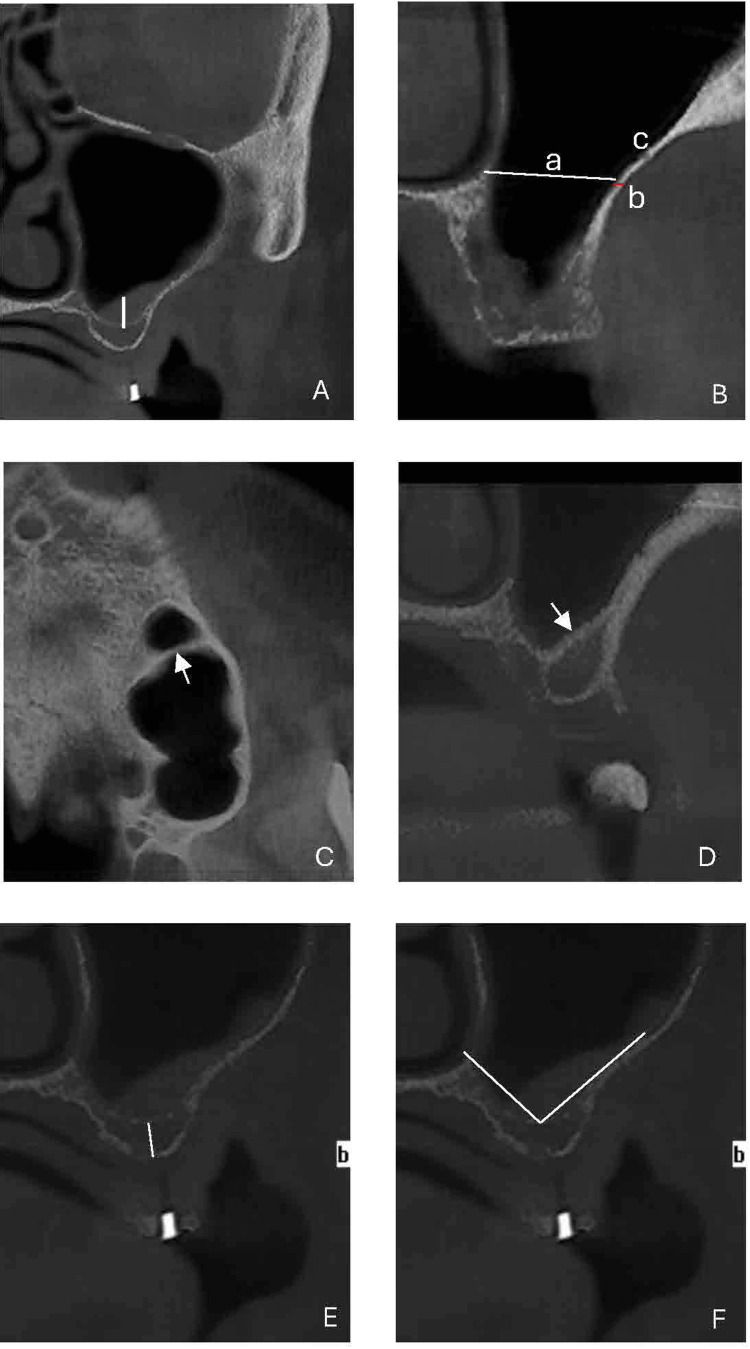
Measurement performed on the CBCT records. Coronal view: (A) Membrane thickness is measured by drawing a line from the deepest point of the convex sinus inferior border to the edge of the mucosal tissue. (B) (a) The width of the maxillary sinus is measured by drawing a horizontal line extending from the medial to the lateral sinus wall at 15 mm height from the residual ridge. (b) Buccal bone thickness measured at the same height. (c) AAA is identified as a radiolucency in the lateral sinus wall. (C) Axial view. (D) Sagittal view: Maxillary septa were identified (Arrows). (E) Coronal view, the residual alveolar ridge height is measured from the line perpendicular to the edentulous area to the inferior border of the maxillary sinus. (F) Coronal view, the buccolingual angle is the angle formed by intersecting the lines parallel to the buccal and vestibular sinus walls.

It is measured from the deepest point of the convex inferior border of the sinus in the coronal view (Figure 1A).

*Sinus Width* 

In the coronal view corresponding to the center of the edentulous area, a horizontal line was drawn from the medial to the lateral wall at a vertical height of 15 mm from the residual ridge (Figure 1Ba)

Buccal Bone Thickness

The lateral bone thickness of the maxillary sinus was measured at 15 mm height from the sinus floor in the coronal view (Figure 1Bb).

Alveolar Antral Artery (AAA) 

The presence of the artery was identified in the coronal view, and the distance between the artery and the alveolar crest was measured (Figure 1Bc).

Maxillary Sinus Septa

Axial and sagittal cuts were used to determine the presence and number of septa (Figures 1C-1D).

Residual Alveolar Ridge Height (RAR)

The RAR measurement was obtained from the coronal view. The bone height was measured on the line perpendicular to the occlusal plane in the edentulous area (Figure 1E).

Angle of the Buccolingual Sinus Wall (BA Angle)

The angle formed by the intersection of the buccal and palatal alveolar wall lines was measured in the coronal view (Figure 1F).

Evaluation of the images

Two periodontal residents (EA and WA) initially evaluated the CBCT images under the supervision of a board-certified periodontist. All examiners were calibrated using a set of 10 CBCT images. Each examiner performed all measurements and repeated the process after two weeks. Inter-examiner and intra-examiner reliabilities (Cohen’s kappa) were 0.998 and 0.997, respectively.

To determine the difficulty of the sinus elevation procedure based on various anatomical factors, a modified scoring system was used following the guideline from Testori et. al [17]. The criteria used are presented in (Table 1).

**Table 1:** Sinus elevation difficulty scoring based on anatomical factors. A modified scoring system was used following the guideline from Testori et al. [17]. Scoring: 1-5 points, simple procedure; 6-9 points, moderate procedure; >10, difficult procedure. AAA, alveolar antral artery

Anatomical factor/Difficulty score	0 point	1 point	2 points
Membrane thickness	1.5-2 mm	0.8-1.49 mm, 2.01-2.99 mm	<0.8 mm, >3 mm
Buccolingual angle	Wide (angle >60°)	Angle within 30°-60°	Narrow (angle <30°)
Sinus width	<15 mm		>15 mm
Buccal bone thickness	<1 mm	1-2 mm	>2 mm
Residual alveolar ridge	>4 mm		<4 mm
Septa	Absence of septa	One septum	Multiple septa
AAA in the augmentation area	Diameter <1 mm	Diameter 1-2 mm	Diameter >2 mm

Statistical analysis

Descriptive characteristics are reported as means and standard deviations for continuous variables, numbers, and percentages for categorical variables. Continuous variables were tested using a paired t-test. While categorical variables were tested using a test of proportions for bivariate analyses. All statistics were performed in STATA software (Version 14.2; Stata, College Station, TX). A significance level of *P* < 0.05 was used.

## Results

This retrospective study evaluated 107 maxillary sinuses in 86 subjects (bilateral sinus evaluation in 17 subjects). The mean age of the study population was 42 ± 13.02 years (range 20-78). Females account for 54.6% (*n *= 47) of the study population (Table 2).

**Table 2:** Clinical characteristics of the study population.

Parameter	Male (*n *= 39)	Female (*n* = 47)	*P*-value
Age	43.59 ± 14.52	41.11 ± 11.67	0.019
Smokers	9	3	
Positive medical history	8	5	
Residual alveolar ridge (RAR)	7.54 ± 3.9 mm	7.32 ± 2.99 mm	0.277
Membrane thickness	2.77 ± 2.74 mm	1.82 ± 1.8 mm	0.011
Buccolingual angle	79.9° ± 17.5°	79.2° ± 17.1°	0.31
Sinus width	14.03 ± 4.46 mm	14.17 ± 3.55 mm	0.34
Buccal bone thickness	1.89 ± 1.1 mm	1.58 ± 0.72 mm	0.11
Alveolar antral artery distance (AAA)	18.4 ± 4.4 mm	13.2 ± 7.15 mm	0.004

Membrane thickness

The average membrane thickness was 2.23 ± 2.3 mm (range 0-10 mm). Males had significantly higher membrane thickness than females (2.77 ± 2.74 mm and 1.82 ± 1.8 mm, *P *< 0.05, respectively). Sixty-seven (62.6%) sinuses had membrane thicknesses within 0-2 mm, while 36 (33.6%) sinuses had membrane thicknesses >2 mm and 16 (15%) sinuses had membrane thicknesses 4 mm or more. Males had significantly more sinuses with a membrane thickness of >4 mm (*P *< 0.05) (Table 3).

**Table 3:** Risk factors associated with sinus augmentation difficulty and complication in the study population. A modified scoring system was used following the guideline from Testori et al. [17]. AAA, alveolar antral artery

Anatomical feature	Favorable	Unfavorable	Risk/Advice
Sinus health	Patent ostium (*n *= 53, 60%)	Non-patent ostium (*n *= 36, 40%)	Medical management before the augmentation procedure
Membrane thickness	1-2 mm (*n* = 28, 26%)	<1 mm (*n *= 39, 36.5%), >4 mm (*n *= 16, 15%)	Higher perforation risk
Buccolingual angle	Moderate or large >65 (*n *= 85, 79.4%)	Small <65 (*n* = 22, 20.6%)	Limited access and visibility
Sinus width	Narrow < 15 mm (*n *= 55, 51.4%)	Wide > 15 mm (*n *= 52, 48.6%)	Wider sinus may require a longer healing time
Buccal bone thickness	1-2 mm (*n = *56, 52%)	<1 (*n* = 22, 20.5%), >2 (*n *= 29, 27%)	Higher perforation risk
Residual alveolar ridge	>4 mm (*n *= 84, 78.5%)	<4 mm (*n *= 23, 21.5%)	Higher perforation risk
Interfering septa	Absent (*n* = 89, 83%)	Present (*n* = 18, 17%), single, multiple	Higher perforation risk depending on the number, direction, and length. Careful evaluation is needed before the augmentation procedure.
AAA in the augmentation area	Not detected (*n *= 59, 55%)	Detected (*n *= 45, 42%)	Increased risk of bleeding. Careful evaluation is needed before the augmentation procedure.

Membrane thickness was related to sinus width (Spearman's rank correlation coefficient rs = 0.23267, *P* = 0.01803) but not to the subject age, BL angle, buccal bone thickness, or interfering septa.

Maxillary sinus septa

Maxillary sinus septa were found in 54 (50.5%) sinuses. Single septa were found in 40 sinuses (74%), followed by two septa in 12 sinuses (22.2%), and three septa in two sinuses (3.7%). However, septa interfering with the sinus augmentation procedure were found in 18 (17%) of all sinuses. The incidence of septa based on gender or age group did not show a significant difference (Table 3).

RAR height

The RAR averaged 7.43 ± 3.37 mm, with a range of 1.25-18.29 mm. RAR was <4 mm in 23 (21.5%) sinuses (Table 3).

Among the sinuses with RAR < 4 mm, six (26%) had interfering septa, one (4.3%) had a membrane thickness of <0.5 mm and one had a membrane thickness of >4 mm.

Buccolingual sinus wall angle

The average mean value of the angle of the maxillary sinus was 79.39° ± 17.16°, revealing a high prevalence of wide-shaped sinuses. The Average mean angle value was 79.9° ± 17.5° for the male group and 79.2° ± 17.1° for the female group (*P *> 0.05) (Table 3).

Sinus width

The average sinus width was 14.09 ± 3.92 mm, with a range of 3.2-23.75 mm. Narrow sinuses (<15 mm) were detected in 55 sinuses (51.4%). However, the difference between males and females was not statistically significant (Table 3).

Buccal bone thickness

The average buccal bone thickness was 1.07 ± 0.90 mm, with a 0.25-4.8 mm range. The difference between males and females was not statistically significant (Table 3). In 29 (27%) sinuses, the thickness was more than 2 mm.

AAA in the lateral wall

AAA can be visualized in the lateral wall of 60 sinuses (56%) and can interfere with the window during lateral sinus augmentation in 45 (42%). On average, the distance of AAA to the alveolar ridge was 17.0 ± 3.69 mm (range 8.55-25.7 mm). AAA was located closer to the alveolar ridge in females than males (13.2 ± 7.15 mm vs. 18.4 ± 4.4 mm, respectively, *P *< 0.05). Only in 5 sinuses (11%), the AAA was located <10mm to the alveolar ridge.

Difficulty score of the MSA procedure

Regarding our population, 89 (83%) sinuses were considered simple MSA procedures and only 18 (16.8%) were considered of moderate difficulty. None of the sinuses evaluated were considered high-risk or inoperable sinuses.

## Discussion

Presurgical evaluation of the maxillary sinus is an essential step during maxillary implant placement treatment planning. This study evaluated anatomical variations affecting the complexity of maxillary sinus augmentation procedure and highlighted the importance of their detection using CBCT. This helps the implant surgeons to anticipate and minimize complications. The study showed that most of the sinuses exhibited features for a simple MSA and only 16.8% of the sinuses are considered moderate difficulty. None of the sinuses evaluated were considered high-risk or inoperable sinuses. Membrane thickness, presence of septa, and AAA were the most complicating factors.

The most common complexity factor was Schneiderian membrane thickness. Although membrane thickness was measured extensively in previous studies; variable results were obtained depending on the technique used to estimate the thickness. However, favorable membrane thickness for MSA was found to be in the 0.8-1.99 mm range and the lowest rate of perforation when the membrane thickness is between 1.5 and 2 mm [18]. Studies have shown a higher rate of perforation with thinner (≤0.5 mm) and thicker membranes (≥3 mm) [19]. The average membrane thickness in this study was 2.23 mm, with males showing a higher incidence of thicker membranes. However, 35% of sinuses had a thickness of >2 mm, and 15% had a thickness of >4 mm. Similar findings were reported by Munakata et al. [8]. Although many factors affect membrane thickness, gender was reported to significantly influence the mean overall thickness of the membrane [8].

Additionally, a thicker sinus membrane correlated with ostium obstruction risk [20]. Sinus healing after MSA depends on adequate draining which could only be achieved if the ostium is patent [18]. Compromised drainage could result in postoperative sinusitis, graft infection, and failure [18]. In this study, 15% of patients had sinus thickness >4 mm (ranges between 4 and 10 mm). Ostium is not always visualized in the CBCT, but a membrane thickness of more than 4 mm is considered pathological and warrants medical attention. In a study conducted by Alzain, it was reported that 52% of Saudi patients seeking dental implants exhibited sinus pathosis, which was presented as a thicker membrane. The majority of these cases were attributed to nonodontogenic factors [21].

The presence of underwood sinus septa leads to technique difficulty during MSA, and it’s not surprising to be correlated to membrane perforation [10]. Septa were found in half of the sinuses evaluated in this study. This is slightly higher than what was reported in previous studies evaluating the Saudi population, which ranged between 25% and 37% [22,23]. Unlike our study, in which we evaluated sinuses where posterior teeth were missing, those other studies evaluated dentate or partially dentate subjects. It is known that the incidence of septa increased with sinus pneumatization due to the loss of posterior teeth [24]. The majority of sinuses contained one septa, while sinuses with multiple septa are not uncommon similar to what was previously reported [10].

Intraosseous AAA was detected in almost half of the study sample. This is similar to previous studies in which intraosseous AAA was detected in 56%-62% of all sinuses [12,25]. However, in only 8.3%, AAA was interfering with the MSA window preparation. In this study, only five sinuses had AAA located less than 10 mm to the alveolar ridge and caused significant interference with an MSA. Variable distance between the AAA location and sinus floor or alveolar ridge was reported [25, 26] with the shortest being at the first molar location [25], or second [26]. Variability in the results could be due to racial differences in the population studied and the inclusion of dentate subjects in the sample. We could not find studies from Saudi Arabia that looked at AAA location and distance in the Saudi population to compare our results. However, like this study, gender differences in the AAA-alveolar ridge distance were previously reported [25]. Injury to AAA during sinus augmentation could result in significant bleeding intra-and postoperatively, complicate the augmentation procedures, and reduce the success rate, in addition to causing significant distress to the patient.

Other factors that we evaluated in the study were sinus width BA angle and buccal bone thickness. Literature has abundant of studies that looked at the role of those factors in complicating MSA [27,28]. A wider sinus width and a larger angle are generally favorable for MSA as they reduce the risk of membrane perforation. On the other hand, a narrower sinus width and a lower angle may require different approaches or techniques to avoid complications [27]. To our knowledge, this study is the first to report on anatomical factors in Saudi patients seeking dental implant treatment. The majority of the sinuses evaluated in this study showed favorable width, BA angle, and buccal bone thickness.

Presurgical evaluation of the maxillary sinus is critical. Radiographic analysis and classification of the sinus to evaluate the type of sinus, risk level, difficulty factors, and best surgical approach is mandatory to reduce the risk of intra and post-surgical complications. Tavelli et al. [18] introduced a difficulty score based on anatomic factors that affect membrane perforation rate. Testori et al. expanded this complexity score to include patient-related factors and categorize the MSA as low, moderate, and high risk for complications [29]. More recently Testori et al introduced the MSA complexity score where MSA difficulty was identified based on a range of anatomical and patient-related factors and maxillary sinus were classified as simple, moderate, or difficult [17]. In this study, we did not evaluate clinical findings so the MSA difficulty score by Testori et al. was modified to include only the anatomical factors to classify the MSA into simple, moderate, or difficult procedures. We found that the majority of sinuses in our sample population were either classified as simple or moderate difficulty risks and were manageable by the available surgical techniques.

The results of this study should be interpreted with caution due to certain limitations. First, the study sample was obtained from a single dental institute, which may not fully represent the broader population. Therefore, the findings may not be generalized to other centers or populations. Additionally, the retrospective nature of the study restricted the evaluation to radiographic features alone, and patient-related factors were not examined. Future research should consider a larger and more diverse sample, as well as incorporate comprehensive patient-related factors.

## Conclusions

Careful presurgical evaluation of the maxillary sinus by CBCT is mandatory before MSA. The findings of this study, which examined a sample of Saudi patients seeking dental implant treatment of the posterior maxilla, demonstrated that the majority of sinuses exhibited a low to moderate risk of complications during MSA. Notably, higher membrane thickness, the presence of septa, and the proximity of the alveolar antral artery were identified as the major significant risk factors for potential complications. These findings underscore the importance of thorough assessment and consideration of these factors to minimize the risk of complications and ensure successful outcomes in MSA.
